# Temporal Characterization of Microglia-Associated Pro- and Anti-Inflammatory Genes in a Neonatal Inflammation-Sensitized Hypoxic-Ischemic Brain Injury Model

**DOI:** 10.1155/2022/2479626

**Published:** 2022-03-02

**Authors:** Maria E. Bernis, Yvonne Schleehuber, Margit Zweyer, Elke Maes, Ursula Felderhoff-Müser, Daniel Picard, Hemmen Sabir

**Affiliations:** ^1^Department of Neonatology and Pediatric Intensive Care, Children's Hospital University of Bonn, Bonn, Germany; ^2^Deutsche Zentrum für Neurodegenerative Erkrankungen (DZNE), Bonn, Germany; ^3^Department of Pediatrics I, Neonatology and Experimental Perinatal Neurosciences, University Hospital Essen, University Duisburg-Essen, Essen, Germany; ^4^Division of Pediatric Neuro-Oncogenomics, German Consortium for Translational Cancer Research (DKTK), Partner site Essen/Düsseldorf, Düsseldorf, Germany; ^5^Department of Pediatric Oncology, Hematology and Clinical Immunology, Medical Faculty, Heinrich Heine University, Düsseldorf, Germany

## Abstract

Hypoxic-ischemic encephalopathy (HIE) mainly affects preterm and term newborns, leading to a high risk of brain damage. Coexisting infection/inflammation and birth asphyxia are key factors associated with intracerebral increase of proinflammatory cytokines linked to HIE. Microglia are key mediators of inflammation during perinatal brain injury, characterized by their phenotypic plasticity, which may facilitate their participation in both the progression and resolution of injury-induced inflammation. The purpose of this study was to investigate the temporal expression of genes associated with pro- and anti-inflammatory cytokines as well as the nucleotide-binding domain, leucine-rich repeat protein (NLRP-3) inflammasome from microglia cells. For this purpose, we used our established neonatal rat model of inflammation-sensitized hypoxic-ischemic (HI) brain injury in seven-day-old rats. We assessed gene expression profiles of 11 cytokines and for NLRP-3 using real-time PCR from sorted CD11b/c microglia of brain samples at different time points (3.5 h after LPS injection and 0, 5, 24, 48, and 72 hours post HI) following different treatments: vehicle, *E. coli* lipopolysaccharide (LPS), vehicle/HI, and LPS/HI. Our results showed that microglia are early key mediators of the inflammatory response and exacerbate the inflammatory response following HI, polarizing into a predominant proinflammatory M1 phenotype in the early hours post HI. The brains only exposed to HI showed a delay in the expression of proinflammatory cytokines. We also demonstrated that NLRP-3 plays a role in the inflammatory resolution with a high expression after HI insult. The combination of both, a preinfection/inflammation condition and hypoxia-ischemia, resulted in a higher proinflammatory cytokine storm, highlighting the significant contribution of acute inflammation sensitizing prior to a hypoxic insult on the severity of perinatal brain damage.

## 1. Introduction

Neonatal hypoxic-ischemic encephalopathy (HIE) is a type of brain injury due to a lack of oxygen and blood flow to the brain during the neonatal period [1]. HIE is a leading cause of neonatal mortality, and it is associated with a variety of life-long morbidities [2, 3]. HIE has an incidence of approximately 1.5 cases per 1000 live births in developed countries and 10-20 per 1000 live births in low- and middle-income countries [4–6]. The etiology of HIE is multifactorial; antenatal infection/inflammation (i.e., chorioamnionitis), hypoxia-ischemia, and various postnatal injurious triggers contribute to the severity of the brain injury and adverse outcome [2, 3, 7–10]. Therapeutic hypothermia (TH) is the current standard treatment for newborns with HIE [4, 11]. TH reduces the risk of death or adverse long-term neurodevelopmental outcome by 15%, leading to improved outcomes following moderate HIE. However, as shown in randomized controlled trials, only 50% of cooled asphyxiated newborns benefit from cooling treatment [4]; and TH has not demonstrated improvement in outcomes related to severe HIE and neonatal encephalopathy in the context of perinatal infection [12–16].

Several studies in animal models, where lipopolysaccharide (LPS) was used as infection simulants, showed that acute infections following inflammation before a second insult such as hypoxia-ischemia (HI) (termed “sensitization”) exacerbate brain injury [15, 17–26]. Previous studies from our laboratory demonstrated that TH, showing significant neuroprotection after HI brain injury in our animal model, is not neuroprotective in our neonatal animal model of inflammation-sensitized HI brain injury [15, 17].

Exposure to multiple inflammatory perinatal triggers contributes to the development of a self-perpetuating cascade of peripheral and cerebral immune-inflammation responses that play a critical role in HI brain injury [27]. Microglia are specialized immune cells of the central nervous system (CNS) [28–30]. During an insult, such as LPS-associated inflammation or HI [31], microglia are rapidly activated, changing their morphology into a motile “amoeboid” state, proliferate, and migrate to the damage regions, where they release a variety of cytokines, chemokines, reactive oxygen species, and excitotoxicity molecules [32, 33]. Studies show that depletion of microglia in models of HIE increases brain injury, indicating the additional essential role of microglia for tissue repair [34, 35]. Two distinct polarization states of activated microglial cells have been discovered. Depending on microenvironmental cues, microglia can rapidly change their phenotype to proinflammatory (type M1) cells or anti-inflammatory (type M2) cells [36, 37]. The polarization of M1/M2 microglia does not seem to follow strict differentiation and can differ depending on the different levels of brain maturity and vulnerability to aggression due to regional and age-specific metabolic needs [38, 39].

The nucleotide-binding domain, leucine-rich repeat protein (NLRP-3) inflammasome, is highly involved in neonatal brain injury either due to LPS or HI [40]. NLRP-3 is responsible for the cleavage of interleukin IL-18 and IL-1beta from its preforms. As shown, the vulnerability of the neonatal brain to LPS or HI is IL-18 and IL-1beta dependent, predominantly leading to microglia activation [41].

We have previously shown in our inflammation-sensitized model of HI brain injury a significant increase in brain area loss and neuronal injury 24 h post HI [42]. Additionally, we have shown that microglia polarize into a predominantly proinflammatory phenotype 24 h post HI, and we showed an increase in the gene expression of NLRP-3 [42, 43]. However, we have only analyzed one time point so far—24 h post HI. The time-dependent expression of pro- and anti-inflammatory cytokines regulated by microglia has yet not been investigated.

Therefore, we used LPS, a component of the cell walls of gram-negative bacteria, and a potent endotoxin, to presensitize the brain and to simulate perinatal infection and inflammation. We hypothesized that the combination of LPS and HI would exacerbate brain injury compared to HI alone, with an early increase in the expression pattern of proinflammatory cytokines as well as the activation of the NLRP3 inflammasome.

## 2. Material and Methods

### 2.1. Animals and Experimental Procedure

**Figure 1:**
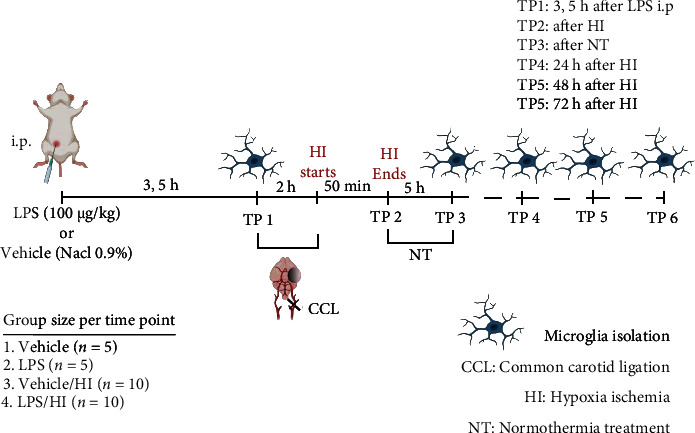
Experimental design: 7-day-old rats (P7) were randomized into 4 different treatment groups. Rats were either injected intraperitoneally (i.p.) with vehicle (NaCl 0.9%) or LPS (100 *μ*g/mL) 3.5 h before exposure to unilateral ligation of the left common carotid artery. Following ligation, rats were exposed to hypoxia treatment (HI) (8% O_2_-36°C) for 50 min before being treated with normothermia (NT) (37°C) for 5 h and thereafter returned to their dam. Different time points were analyzed. TP1 is 3.5 h before ligation. TP2 is immediately after HI. TP3 is immediately after NT. TP4, TP5, and TP6 are 24, 48, and 72 h after HT, respectively. The brain was extracted and microglia isolated via magnetic cell sorting for CD11 b/c-positive cell. Figure created with http://BioRender.com.

All animal experiments were performed in accordance with the Animal Protection Committee of the North Rhine-Westphalia State Environment Agency (LANUV), Germany, and following the ARRIVE guidelines. The study was performed using 7-day old (P7) Wistar rat pups of both genders in all our experiments. All pups were kept at the central animal laboratory of the Deutsche Zentrum für Neurodegenerative Erkrankungen (DZNE) Bonn, Germany, with a 12 : 12 h dark/light cycle at an environmental temperature of 21°C with food and water ad libitum. As previously described, all animals for each treatment were randomized across litter, sex, and weight before the experiments commenced, and all following experiments and analysis were performed by observers blinded to the different treatments [15, 17]. A total of 244 animals were used (129 females and 115 males) and randomized to different treatment groups. 160 rat pups survived our inflammation-sensitized HI insult. Mortality was highest in the LPS/HI group as it has been expected and reported [15, 17, 42]. Four groups were used and brains analyzed at different time points: vehicle (*n* = 5 per time point), LPS (*n* = 5 per time point), vehicle/HI (*n* = 10 per time point), and LPS/HI (*n* = 10 per time point). Temperature was monitored in “sentinel” rat pups not allocate to the different treatment groups during experimental procedures. During all the experiment, temperature of the rat pups was controlled by the sentinel pup via a rectal probe (IT-21, Physitemp Instruments, Clifton, NJ, USA) controlling a mat (CritiCool, MTRE, Yavne, Israel). The sentinel pup kept the nesting temperature of P7 rat pups [19] or treatment temperatures during experiments (see below). All rats from the LPS and LPS/HI group received a single intraperitoneal (i.p.) injection of LPS solution (Escherichia coli O55:B5, Sigma; 0.1 mg/kg). The vehicle and vehicle/HI groups received a single i.p. injection of saline (NaCl 0.9%). After a delay of 3.5 h post injection, while animals were kept with their dams, and the vehicle/HI and LPS/HI animals were exposed to our model of HI brain injury as previously described [15, 17]. Briefly, under general isoflurane anesthesia, the left common carotid artery was ligated and cut. Within 3 h, the pups were subjected to 8% O_2_ for 50 min at a rectal temperature (*T*_rectal_) of 36°C, resulting in mild HI brain injury [15, 17]. Immediately after HI, pups were kept at *T*_rectal_ of 37.0°C for 5 h, representing the normothermia treatment group in our previous experiments [15–17]. Following the treatment period, the pups were returned to their dam. The pups were sacrificed at different time points (Figure 1). For all the pups at the first time point (TP1; see Figure 1), no ligation was performed, nor exposure to HI.

### 2.2. Immunohistochemistry

For histological analysis, the pups were sacrificed at TP4 (24 h after HI). Following transcardial perfusion with phosphate-buffered saline (PBS), followed by 4% paraformaldehyde (Sigma-Aldrich), the brains were postfixed in 4% paraformaldehyde overnight at 4°C and embedded in paraffin. Immunohistochemistry was performed as previously described [44, 45]. We used TP4 for our immunohistochemistry since at TP5 and TP6 (48 and 72 h post HI, respectively), the cortex and hippocampal areas in the LPS/HI were severely affected (data not shown). After deparaffinization, 10 *μ*m coronal sections (−3.8 ± 0.7 mm from bregma) were rehydrated. Antigen retrieval was performed in preheated PBS 1x for 7 min following permeabilization with 0.1% Triton X-100 for 30 min at room temperature. After blocking with 20% normal goat serum in PBS 1x (Invitrogen, Germany), slices were incubated with primary antibodies overnight at 4°C followed by appropriate secondary antibody incubation for 1 h at room temperature. Both primary and secondary antibodies were diluted in 0.7% Carrageenan solution with 0.02% NaN_3_ solution in PBS 1x. The sections were counterstained with 4,6-diamidino-2-phenylindole (DAPI) (Invitrogen, Germany). Microglia activation was detected by using ionized calcium-binding adaptor molecular 1 (Iba1) (rabbit polyclonal, 1 : 200, Cat. N°: 019-19741 (RRID: AB_839504), Wako, Germany), anti-Caspase-1 (p20) (mouse monoclonal, 1 : 500, Cat. N°: AG-20B-0042-C100 (RRID: AB_2755041), AdipoGen), and anti-NLRP-3 inflammasome (rabbit polyclonal, 1 : 200, Cat. N°: ab214185 (RRID: AB_2819003), Abcam, Germany) on sections of P8 rat pups. Because Iba-1 and NLRP-3 antibodies have the same host, we proceeded with sequential immunostaining. Immunohistochemistry were visualized by fluorescence microscopy AxioScan Z.1 (Zeiss, Germany), using a 20x objective. The images were analyzed using Zen 3.1 (Blue edition, Zeiss, Germany) and ImageJ. From each ipsilateral hemisphere, we analyzed the hippocampal areas (CA1, CA2, CA3, and CA4), ventroposterior thalamus nucleus, subthalamic nucleus, basolateral amygdaloid nucleus, and caudate putamen. We did not select the cortex area because of the amount of damage present. We selected the hippocampal regions (CA1, CA2, CA3, and CA4) as the most representative area due to the high expression of the markers used (Iba-1, Caspase-1, and NLRP-3). For Iba-1, we took a single image at 20x magnification to visualize the morphology of the microglia cells using a ZOE Fluorescent Cell Imager microscope (Bio-Rad, Germany). The contralateral side did not show differences in staining.

### 2.3. Magnetic Activated Cell Sorting (MACS) of CD11 b/c-Positive Microglia

For microglia isolation, we proceeded to do magnetic cell sorting of CD11 b/c-positive microglia from the ligated brain hemispheres. Pups were sacrificed at different time points (Figure 1). To analyze the different alterations in phenotype polarization of microglia, we specifically isolated CD11 b/c-positive microglia ex vivo from rat brains at the previously mentioned time points. Due to the limitation of the technique regarding the amount of isolated cells, the severity of damage on the ipsilateral brains (especially at TP5-6), and because of the high mortality in our LPS/HI group, we proceeded to isolate and pool the brains for each condition and time point in order to get a workable amount of microglia cells with the highest yield required for an optimal RT-PCR of the full set of cytokines selected for the study. For vehicle and LPS, 5 full brains were pooled from each separate condition since they were not exposed to HI. For vehicle/HI and LPS/HI, a total of 10 ipsilateral hemispheres (most affected hemisphere after HI; [15, 17]) were pooled per condition and time point. The brains were mechanically dissociated in HBSS 1x cold buffer. Myelin-removal beads were used following distributors instructions followed by magnetic separation on LS columns. For MACS, the obtained cell mixtures were washed with MACS buffer (PBS 1x containing 0.5% BSA and 0.2 mM EDTA) and incubated with anti-CD11b/c coupled microbeads (Miltenyi Biotec, Germany) followed by magnetic separation on MS columns of the mini-MACS magnetic separation kit (Miltenyi Biotec, Germany) following distributor instructions. The total effluent (negative fraction), after removal of the column CD11 b/c-positive microglia, was eluted in a volume of 1 ml MACS buffer. To investigate the purity of the magnetically separated cells, a small volume of the positive eluate was analyzed via flow cytometry (data not shown). The rest of separated positive CD11 b/c cells was used for real-time PCR as a unique sample for each treatment in each time point.

### 2.4. Real-Time PCR

RNA from isolated microglia was generated following the distributor instructions from RNeasy Mini Kit (QIAGEN, Germany). First strand complementary DNA was synthesized using 2 *μ*g of total RNA and TaqMan reverse transcription reagents (Applied Biosystems/Thermo Fisher Scientific, USA). The 96-well optical reaction plates were used to perform the PCR amplification. 40 cycles (each cycle at 94°C for 15 s and 60°C for 1 min) were done using the StepOnePlus Real-Time PCR system (Applied Biosystems/Thermo Fisher Scientific, USA). Analysis was performed at different time points (TP1, TP2, TP3, TP4, TP5, and TP6) in our 4 predefined groups (vehicle, LPS, vehicle/HI, and LPS/HI). The PCR results of pro- and anti-inflammatory cytokines were quantified by fluorogenic reporter oligonucleotide probes. Pro- and anti-inflammatory markers as well as gene expression of the inflammasome cryopyrin (NLRP-3), including as well *Caspase-1* and *IL-18* used in this study, are listed in Table 1 and were purchased from Thermo Fisher Scientific, Germany. *Beta*-actin was used as housekeeping gene. The results were normalized to the vehicle group. Generally, real-time PCR and detection were performed in duplicates (2-technical replicates). Target gene expression was quantified according to the 2^*ΔΔ*CT^ method [46]. Fold change was used as “magnitude scale” to classify the extend of gene expression for the pro- and anti-inflammatory effects after the different treatments over the times analyzed. Figures were plotted as each condition per separate, including all time points together for the purpose of a better overview of the effect over time after the different treatments.

### 2.5. RNA Sequencing and Gene Set Analysis

RNA sequencing and gene set analysis were performed as previously described [43]. Ingenuity Pathway Analysis was performed on the significant genes comparing a condition of interest to other conditions (i.e., hypoxia alone samples vs LPS/HI alone samples). The data from upstream regulators were then plotted as a volcano plot (*x*-axis activation, z-score, *y*-axis, and -log10 *p* value). From 549 gene expression profiles for different regulators expressed in Veh/HI and LPS/HI at 24 h post HI, we preselected only upstream regulators, which are cytokines expressed in both condition. Highlighted in red are the most significant cytokines expressed in both groups, while those cytokines present only in the LPS/HI condition were highlighted in yellow. For our study, the following 11 cytokines were used: proinflammatory M1: *IL1-beta*, *IL-6*, *IL-12*, *iNOS*, and *TNF-alpha* and anti-inflammatory M2*: Arg-1*, *CD206*, *CCL11*, *IL-4*, *LIF*, and *TGF-beta*) [47–49].

### 2.6. Statistical Analysis

GraphPad Prism 6 (GraphPad Software, United States) was used to analyze and plot the data. For the RNA sequencing and gene set analysis, a *t*-test was performed to determine differential expression at the gene level (*p* < 0.05, fold change ± 2).

## 3. Results

### 3.1. Transcriptomic Profiling of Presensitized Microglia after Hypoxia-Ischemia

**Figure 2:**
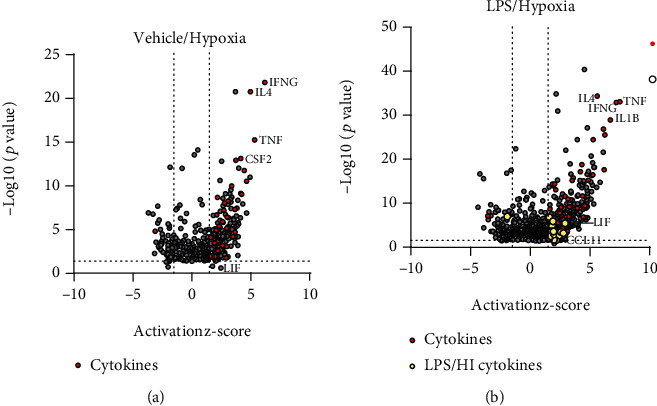
Significant upregulation of several cytokine genes following inflammation-sensitized HI injury. Positive CD11 b/c microglia were used for transcriptome analysis (RNA sequencing). Volcano plot (*x*-axis activation, z-score, *y*-axis, and -log10 *p* value) representing all upstream regulators in the LPS/HI and vehicle/HI groups (gray), where upstream cytokine regulators are highlighted in red for vehicle/HI and LPS/HI group (a–b) and cytokines only present in the LPS/HI group are highlighted in yellow (b).

Previous studies from our group demonstrated both a pro- and anti-inflammatory cytokine response 24 h post HI in our inflammation-sensitized HI model [43]. To further investigate the specific phenotype polarization of microglia in our study, we performed transcriptomic profiling of microglia cells in our model 24 hours post HI. At that time point, the cytokine profile showed a high regulation for genes with relevant function in microglia activation [42, 43]. After RNAseq, we focused on a gene set of microglia cytokines, as this clustered gene set showed the most significant genes upregulated in the vehicle/HI and LPS/HI groups, as illustrated in a volcano plot (Figures 2(a) and 2(b)). From the full set of gene expression in both conditions (see Material and Methods), we highlighted in red the upstream regulators, which are cytokines presented in both groups, while in yellow, only those expressed in the LPS/HI group are presented. In the present study, a total of 11 most significant genes from the cytokines cluster were selected in base of their function in microglia activation and inflammation [25, 31, 50, 51]. For our study, the following cytokines were used: proinflammatory M1: *IL1-beta*, *IL-6*, *IL-12*, *iNOS*, and *TNF-alpha* and anti-inflammatory M2*: Arg-1*, *CD206*, *CCL11*, *IL-4*, *LIF*, and *TGF-beta* [47–49].

### 3.2. Time Dependency of Proinflammatory Genes in Microglia after LPS-Sensitized Hypoxic-Ischemic Brain Injury

**Figure 3:**
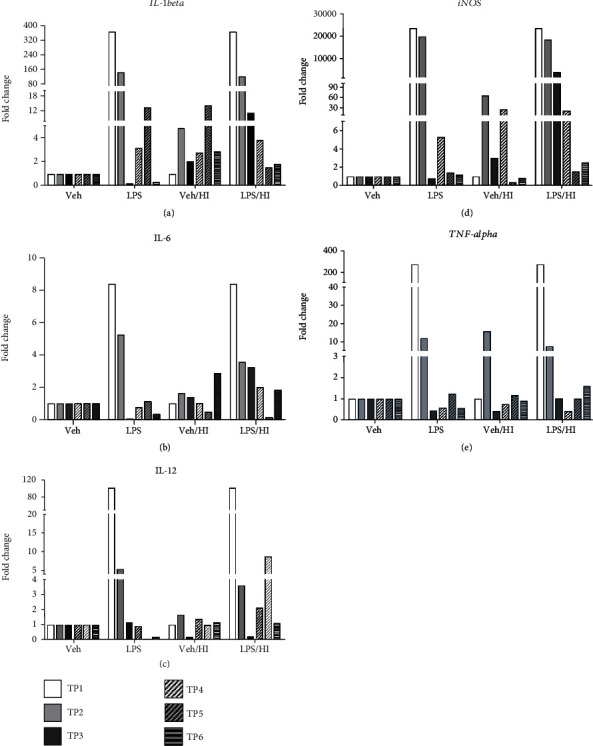
Proinflammatory cytokines expression of CD11 b/c microglia before and after inflammation-sensitized HI brain injury. A substantial upregulation immediately after inflammation-sensitized LPS compared to the vehicle group at TP1 was observed for all cytokines analyzed (3.5 h after i.p. LPS injection). (a) *IL-1*beta showed a remarkable upregulation at TP2, TP3, and TP5, with a slight decrease in the gene expression through TP4 and TP6. Only TP2, TP3, and TP5 showed a prevailing regulation of its expression in the group LPS/HI compared to the vehicle/HI. (b) *IL-6* showed a similar upregulation pattern as *IL-1*beta with a slight increase after TP6, with a remarkable upregulation in the LPS/HI group compared to the vehicle/HI at TP2, TP3, and TP6. (c) *IL-12* showed upregulation at TP2 and TP5 in the LPS/HI group compared to the vehicle/HI. (d) *iNOS* showed upregulation from TP2 to TP3 for vehicle/HI and LPS/HI groups. No changes in gene expression after TP5 were observed. (e) *TNF-alpha* showed a similar expression pattern as iNOS, with an increased at TP2, even for the LPS/HI compared to the vehicle/HI. No changes in the gene expression were found in the following TPs. Group sizes: vehicle *n* = 5, LPS *n* = 5, vehicle/HI *n* = 10, and LPS/HI *n* = 10 (microglia from the whole brains were pooled for vehicle or LPS, while 10 ipsilateral hemispheres were pooled for vehicle/HI or LPS/HI).

To assess the inflammatory response in our LPS presensitized model before and after HI exposure, we compared gene expression profiles associated with proinflammatory cytokines in sorted CD11b/c microglia cells from pooled full brains from the vehicle and LPS alone groups, while the ipsilateral hemisphere brains were pooled and used for the vehicle/HI and LPS/HI groups [52]. We analyzed and quantified the gene expression levels of the following proinflammatory cytokines: *IL-1beta*, *IL-6*, *IL-12*, *iNOS*, and *TNF-alpha*. To determine the degree of inflammation in our model before exposure to HI, we analyzed microglia-associated cytokines before animals underwent carotid ligation and hypoxia. Our data showed a clear increase in the expression level for all the proinflammatory cytokines analyzed at TP1 (3.5 h before HI) (Figures 3(a)–3(e)), where *iNOS* showed an increase with a magnitude of 20.000-fold changes compared to the vehicle group, followed by a 100-fold change for *IL1-beta*, *IL-12*, and *TNF-alpha* compared to the vehicle group, while *IL-6* showed still an 8-fold change compared with the vehicle group.

At TP2, we observed a prevailing increase in the expression levels for all the proinflammatory cytokines analyzed. The LPS/HI group showed a clear upregulation compared to the vehicle group for all the proinflammatory cytokines analyzed (Figures 3(a)–3(e)). Interestingly, the LPS/HI group showed also a substantial increase in the expression level compared to the vehicle/HI group. We observed for *iNOS* a 10.000-fold change in its expression level compared to the vehicle/HI group (Figure 3(d)), followed by a 100-fold change for *IL-1beta* compared to the same groups (Figure 3(a))*. IL-6*, *IL-12*, and *TNF-alpha* showed less increase in their gene expression (Figures 3(b), 3c, and 3(e)). Additionally, LPS treatment alone led to a substantial increase in the expression for all the cytokines tested compared to the expression level of the vehicle group (Figures 3(a)–3(e)): fold change: *iNOS* > 10.000, *IL* − 1*beta* > 100, *TNF* − *alpha* > 10, *IL-6*, and *IL* − 12 > 5). The vehicle/HI group showed a slight increase in its expression, showing a 10-fold change for *TNF-α* compared to the vehicle group (Figure 3(e)). This observation was less pronouncing when we compared the vehicle/HI group to the LPS/HI group (Figure 3(e)).

At TP3, *iNOS* showed a 100-fold change in the LPS/HI group compared to the vehicle group (Figure 3(e)), while *IL-1beta* and *IL-6* showed a less pronounce change in their expression level (10-fold change) in the LPS/HI group compared to the vehicle group (Figures 3(b) and 3(d)). The expression level of *IL-1beta* and *IL-6* in the LPS group showed a substantial decrease compared to the LPS/HI group (Figures 3(a), 3(b), and 3(d)). Interestingly, we observed an increase in the LPS/HI group compared to the vehicle/HI group, where *IL-1beta* showed a 1000-fold change compared to the vehicle/HI. *IL-6* and *iNOS* showed a slight increase of 10-fold change (Figures 3(a), 3(b), and 3(e)). *IL-12* and *TNF-alpha* showed a decrease in the gene expression for all treatment groups compared at TP3 (Figures 3(c) and 3(e)). At TP4, *TNF-alpha* and *IL-12* showed no changes in their expression level in the different groups analyzed (Figure 3(a), 3(c), and 3(e)). However, *IL-1beta* and *iNOS* showed a slight upregulation in all the groups compared to the vehicle group alone (Figure 3(a) and 3(d)). At TP5, *IL-1beta* showed a slight increase in the LPS and vehicle/HI groups compared to the vehicle alone and LPS/HI group (Figure 3(a), fold change > 10). *IL-6* showed a downregulation in the expression level for LPS/HI compared to LPS (Figure 3(b)); and it was the only proinflammatory cytokine that at TP6 showed a slight increase on the vehicle/HI and the LPS/HI groups compared to the vehicle group (Figure 3(b)).

Our results at TP1 demonstrate that LPS sensitized microglia to a major inflammatory status and further following HI resulted in a constant inflammatory status that lasted over the time points studied, while the brains not presensitized showed a later regulation in their proinflammatory expression pattern.

### 3.3. Time Dependency of Anti-Inflammatory Genes in Microglia after LPS-Sensitized Hypoxic-Ischemic Brain Injury

**Figure 4:**
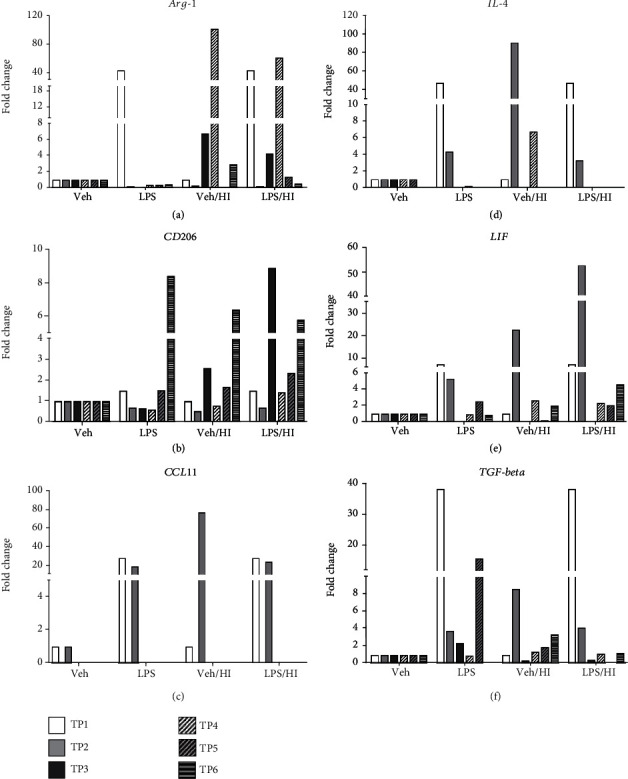
Anti-inflammatory cytokines expression of CD11 b/c microglia after inflammation-sensitized HI brain injury. A substantial upregulation immediately after inflammation-sensitized LPS compared to the vehicle group at TP1 was observed for *Arg-1* (a), *CCL11* (c), *IL-4* (d), and *TGF-beta* (f) (3.5 h after i.p. LPS injection). (a) *Arg-1* only showed upregulation at TP4 for the LPS/HI group compared to the vehicle/HI group. (b) *CD206* showed upregulation at TP3 and at TP6, where only at TP3, LPS/HI showed a prevailing regulation against vehicle/HI group. (c–f) *CCL11*, *IL-4*, *LIF*, and *TGF-beta* only showed upregulation in gene expression immediately after HI at TP2. However, only *CCL11*, *IL4*, and *LIF* showed a change in the expression pattern between LPS/HI and vehicle/HI at TP2. Group sizes: vehicle *n* = 5, LPS *n* = 5, vehicle/HI *n* = 10, and LPS/HI *n* = 10 (microglia from the whole brains were pooled for vehicle or LPS, while 10 ipsilateral hemispheres were pooled for vehicle/HI or LPS/HI).

The balance between pro- and anti-inflammatory mediators and their interactions is known to determine the magnitude of the inflammatory reaction. We assessed gene expression profiles associated with anti-inflammatory cytokines from sorted CD11b/c microglia from pooled full brains from the vehicle and LPS groups alone, while the ipsilateral hemisphere of brains were pooled and used from the vehicle/HI and LPS/HI groups [52]. As it was mentioned above, the degree of inflammation in our model before exposure to HI was analyzed from microglia-associated cytokines before animals underwent carotid ligation and hypoxia. Interestingly, the expression levels of classical anti-inflammatory cytokines (Figure 4(a)–4(f)) did not show as high expression levels as it was observed for the proinflammatory cytokines. At TP1, an increase for *Arg-1*, *IL-4*, and *TGF-beta* with a 40-fold change expression compared to the vehicle group alone was observed, while *CCL11* and *LIF* showed a slight increase (fold change > 20 and fold change > 10, respectively) compared to the vehicle group. However, *CD206* did not show any change in its expression level at this time point (Figure 4(b)).

As described before for TP1, a high gene expression for some anti-inflammatory cytokines immediately after LPS exposure demonstrates that microglia plays a dual role in the resolution of inflammation not only by the expression of proinflammatory cytokines but also by the regulation of anti-inflammatory cytokines [36, 53, 54]. At TP2, only *L1F* (Figure 4(e)) showed a 50-fold change increase in its expression level in the LPS/HI group compared to the vehicle group alone, while vehicle/HI group showed a 20-fold change increase compared to the vehicle group alone. *CCL11* showed a slight increase in its expression for the LPS/HI and LPS groups compared to the vehicle group alone (Figure 4(c), fold change > 20) and a 50-fold change in its expression in the vehicle/HI group compared to the vehicle group alone. *IL-4* showed a 50-fold change increase in its expression levels only for the vehicle/HI group (Figure 4(b)). A slight upregulation was observed for TGF-beta with a 10-fold change for the same groups (Figure 4(f)). *CD206* showed minimal changes in its gene expression at TP3 and TP6 in the LPS/HI group compared to the vehicle, LPS, and Veh/HI groups (Figure 4(b), fold change < 10). On the other side, at TP4, *Arg-1* showed a slight upregulation with a 50-fold change for the LPS/HI and vehicle/HI groups compared to the vehicle and LPS groups (Figure 4(a)). *TGF-beta* only showed a change at TP5 with a substantial downregulation in its gene expression for the LPS/HI group compared to the vehicle group (Figure 4(f)), while the LPS group compared to the vehicle group showed a moderately upregulation in its gene expression (Figure 4(f)).

### 3.4. Activation of the NLRP3 Inflammasome in Microglia after LPS-Sensitized Hypoxic-Ischemic Brain Injury

**Figure 5:**
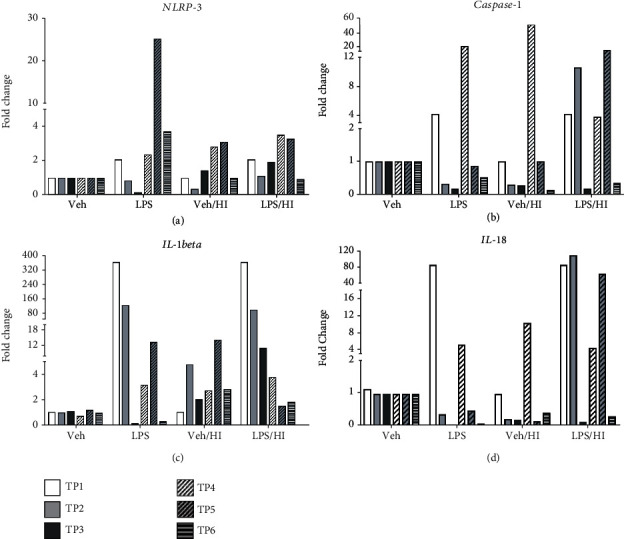
*NLRP-3*, *Caspase-1*, *IL-1beta*, and *IL-18* gene expressions of CD11 b/c microglia after inflammation-sensitized HI brain injury. A substantial upregulation immediately after inflammation-sensitized LPS compared to the vehicle group at TP1 was observed for *Caspase-1* (b), *IL-1beta* (c), and *IL-18* (d) (3.5 h after i.p. LPS injection). (a) NLRP-3 gene expression showed upregulation from TP3 to TP6. At TP5, a substantial increase in the vehicle/HI group compared to the LPS/HI group was observed. (b) *Caspase-1* showed a similar activation pattern as *NLRP-3* with upregulation already at TP2 for the LPS/HI group compared to the other groups. (c) *IL-1beta* and (d) *IL-18* showed an upregulation over the time analyzed and prevailing activation comparable to *NLRP-3* gene expression. Group sizes: vehicle *n* = 5, LPS *n* = 5, vehicle/HI *n* = 10, and LPS/HI *n* = 10 (microglia from the whole brains were pooled for vehicle or LPS, while 10 ipsilateral hemispheres were pooled for vehicle/HI or LPS/HI).

As NLRP-3 mediates Caspase-1 activation and the secretion of IL-1beta/IL-18; in both proinflammatory cytokines [55], leading to microglia activation, we analyzed *NLRP-3*, *Caspase-1*, *IL-1beta*, and *IL-18* gene expressions in our model at early and later time points after HI. We did not find any activity of the inflammasome in microglia exposed to LPS before HI injury (Figure 5(a)). At TP3, *NLPR-3* (Figure 5(a)) showed a slight upregulation in the vehicle/HI and LPS/HI groups compared with the vehicle group (Figure 5(a)). We previously showed a significant upregulation in *NLRP-3* gene expression 24 h post HI from the LPS/HI group compared to the vehicle group and a slight significant gene expression between vehicle/HI compared to the vehicle group [42]. In the present study, LPS, vehicle/HI, and LPS/HI groups showed a 5-fold change increase compared to the vehicle group at TP5, while a 20-fold change increase in gene expression between the LPS group compared to the LPS/HI group was observed. Interestingly, *NLRP-3* expression maintained high in the LPS group at TP6 compared to the vehicle and LPS/HI groups (Figure 5(a)), while no changes were observed for the other treatment groups.

*Caspase-1* showed a slight increase in its expression in the LPS groups compared with the vehicle at TP1. However, a 10-fold change was observed in the LPS/HI group compared with the vehicle at TP2. Interestingly, at TP4, LPS and vehicle/HI showed a 20-fold change increase compared to the vehicle alone, while LPS/HI showed less increment of 10-fold change. We also observed that vehicle/HI showed an expression level of 10-fold change compared to the LPS/HI group (Figure 5(b)).

Only at TP5, LPS/HI group showed a 20-fold change compared to the other groups. TP6 did not show any change in gene expression for all the four groups analyzed. The timing observed for the gene expression for *Caspase-1* followed a pattern associated with the pattern of the *NLRP-3* inflammasome. The analysis for *IL-1beta* (previously described above) indeed showed similar patterns of activation as the *NLRP-3* inflammasome (Figure 5(c)). The analysis of *IL-18* showed similar time point expression as it was observed for *NLRP-3* and *Caspase-1*. An increase of 80-fold change was observed at TP1 for the LPS group compared to the vehicle group (Figure 5(d)), while LPS/HI showed an increase of 80-fold change compared to the vehicle group as well as to the vehicle/HI group at TP2. At TP3, we did not observe major changes in its expression. However, at TP4, LPS/HI, vehicle/HI, and LPS showed an increase compared to the vehicle group alone (Figure 5(d)). Only LPS/HI showed an 80-fold change increase compared to the vehicle/HI, LPS, and vehicle groups at TP5. We did not observe major changes at TP6 for any of the groups analyzed.

### 3.5. Hippocampal NLRP3 Inflammasome and Microglia Activation after LPS-Sensitized Hypoxic-Ischemic Brain Injury

**Figure 6:**
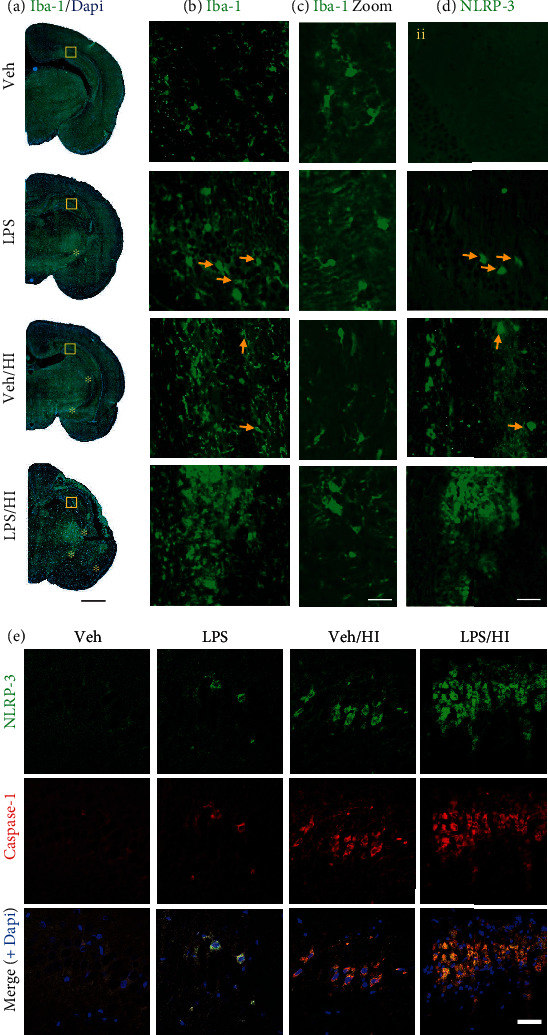
Immunohistochemistry of brain slices (bregma -3.8) at TP4. (a–c) Microglia marker Iba-1 in green and nucleus marker Dapi in blue. (d) NLRP-3 in green. (a) We observed at TP4 a severe lesion in the LPS/HI group compared to the vehicle or LPS groups alone. Yellow squares represent the hippocampal area analyzed in (b) and (d). Yellow asterisks represent other areas with immunopositive staining for Iba-1 and NLRP-3. We observed for the LPS/HI group, NLRP-3-positive staining in the ventroposterior thalamus nucleus, subthalamic nucleus, basolateral amygdaloid nucleus, and caudate putamen, while for the vehicle/HI group, positive staining was only found in the ventroposterior thalamus nucleus and subthalamic nucleus. LPS group alone only showed positive NLRP-3 staining in the ventroposterior thalamus nucleus. (b) Iba-1 staining of the hippocampal area showed an increase in microglia cells with an increase in microgliosis in the LPS/HI group. (c) Magnification of the area in (b), showing the degree of polarization in microglia cells, with a clear, ramified morphology (inactivated microglia) in the vehicle group, to an amoeboid morphology (activated microglia) in the LPS/HI group. (d) NLRP-3-immunopositive staining of the hippocampal area with a remarkable increase of NLRP-3-positive cells in the same area where a strong microgliosis was observed in the LPS/HI group. Yellow arrows point cells positive for Iba-1 and NLRP-3. (e) Double-immunostaining shows colocalization in the hippocampus area for NLRP-3 (green) and Caspase-1 (red) and merge (+nuclear marker, Dapi in blue), with an increase in the amount of positive staining in the LPS/HI group. Scale bar: (a) 500 *μ*m, (b, d–e) 100 *μ*m, and (c) 20 *μ*m.

We previously demonstrated that the upregulated NLRP3 expression 24 h after HI (TP4 in our study) was particularly significant in the hippocampus and cortex of the rats from the LPS/HI group compared to the vehicle group [42]. Using immunohistochemistry staining for NLRP3 and the microglia marker Iba-1 in brain slices from TP4 (Figure 6(a), yellow square (area analyzed in b, c, and d), and Figures 6(b)–6(d)), we showed a strong positive staining in the hippocampus area for the LPS/HI, vehicle/HI, and LPS compared to the vehicle group. We observed severe cortical and hippocampal injury in the ipsilateral side of the brain in the LPS/HI group, which corroborated our observation from the previous work on 7-day postinsult brains with a predominant lesion for the same group [15, 17]. We observed a strong staining of Iba-1 and NLRP3 in the 4 fields from the hippocampus area (CA1, CA2, CA3, and CA4), ventroposterior thalamus nucleus, subthalamic nucleus, basolateral amygdaloid nucleus, and caudate putamen in the LPS/HI group (Figure 6(a) (yellow asterisk) and Figure 6(b)) with a clear staining for activated microglia (Figure 6(c)). The vehicle/HI group showed the most reduced area with Iba-1-positive staining (Figure 6(b), yellow arrows), with a variety of microglia polarization (Figure 6(c)) and much less positive staining for NLRP-3 in the CA4 hippocampus, ventroposterior thalamus nucleus, and subthalamic nucleus (Figure 6(c), yellow arrows). The LPS group showed few Iba-1-positive cells in a less activated state (Figure 6(b)–6(c), yellow arrows) and few positive NLRP-3 cells in the hippocampus, especially areas CA2 and CA4, and in the ventroposterior thalamus nucleus (Figure 6(d), yellow arrows). The vehicle group showed few Iba-1-positive microglia cells with a more ramified morphology and no positive staining for NLRP-3 (Figures 6(b)–6(d)). Due to the severity of the lesion after HI, we could not analyze the cortex area at TP 5. Most of the positive NLRP-3 staining were in the same or proximal to niches of positive microglia cells staining (Figure 3(d), yellow square and yellow arrows). However, we observed NLRP-3-positive staining in other cells, likely hippocampal neurons or motor neurons (data not shown). We also observed in immunohistochemistry from brain sections from TP4 a strong colocalization between NLRP-3 inflammasome and Caspase-1 in the hippocampus area (Figure 6(e)). LPS/HI and vehicle/HI showed a strong staining for NLRP-3 and Caspase-1 in the 4 field from the hippocampus, while LPS group showed few immunopositive cells for NLRP-3 and Caspase-1 (Figure 6(e)). We did not observe immunostaining in the vehicle group.

## 4. Discussion

The risk of developing HIE is significantly increased with the combination of preinfection/inflammation and a potential birth asphyxiating condition, compared to either alone [8, 56–60]. The mechanism by which preinflammation increases brain vulnerability to HI is complex.

A number of observational studies suggest an increased risk of neurodevelopmental impairment following infection/inflammation in newborns [8, 10, 23, 49]. Here, we demonstrate a biological basis possibly explaining the severe adverse outcome in inflammation-sensitized asphyxiated neonates. It is commonly believed that similar to the initiation of inflammation, resolution of inflammation is an active process in which inflammation-resolving cells like microglia and their cytokines are pivotal for the termination of the inflammatory response [61]; however, as much as inflammation is a pivotal process in fighting off many threatening conditions, when it is unresolved, it forms the basis of a wide range of persistent/chronic diseases and secondary damage mediated by the inflammatory response constantly disrupting the return to homeostasis [61].

In the present study, we used bacterial LPS; a component of the cell walls of gram-negative bacteria. Although gram-positive organisms are the most common cause of antenatal/postnatal infection in high resources settings, gram-negative infection/inflammation is increasing in frequency in the low- and middle-resource settings, surpassing gram-positive organisms as the leading causative pathogen in neonatal infection/inflammation associated HIE [62, 63]. In models of LPS sensitization, the mechanism mainly starts with the activation of toll-like receptor 4 (TLR4) which triggers many self-perpetuating pathways, among them are microglia activation/proliferation and release of proinflammatory cytokines [31, 64]. The Vannucci model is the most established animal model for newborn HI brain injury in rodents that led to translational clinical trials and the establishment of TH to reduce mortality and morbidities following perinatal asphyxia [65]. However, clinically up to 50% of all cooled newborns from the large randomized controlled trials did not benefit from cooling therapy [4]. It has been demonstrated that the beneficial effect of TH depends on the time window during which the treatment starts after an insult. Immediately after injury or with a delay of not more than 6 h, TH is neuroprotective after moderate HI; however, the neuroprotective effect is unfavorable when the treatment is delayed or even after a severe insult [16, 66, 67]. Currently, it has been shown that TH is not beneficial in the setting of low- and middle-income countries and strengthens our findings of lack of TH neuroprotection in our LPS-sensitized HI rodent model [14, 15, 17]. However, the molecular mechanism by which LPS presensitization occurs and why HT is not beneficial in particular cases are not known yet.

Previous publications from our laboratory demonstrated a high expression of proinflammatory cytokines and the NLRP-3 inflammasome only at the 24 h time point after the insult in our LPS presensitized model of HI brain injury [42, 43]. However, the time-dependent temporal expression of pro- and anti-inflammatory cytokines in this model is of main interest, as it might give us the possibility to understand why TH is not neuroprotective in this model and at what time points potential alternative treatments might be beneficial.

We propose that microglia play a key role in the severity of brain injury following LPS-sensitized HI [15, 43], with a predominant proinflammatory (M1) and anti-inflammatory (M2) phenotype in the first hours after LPS, exacerbating the microglia response after a mild HI injury compared to HI alone. However, the effect observed in a not presensitized brain showed a delay in the inflammatory resolution by microglia cells. We highlight the involvement of the NLRP3 inflammasome in the inflammatory process of our brain injury model preexposed with LPS and following HI, with a remarkable lesion at early time points that could explain the severity of the LPS-sensitized HI brain injury observed at 7-day post insult [15, 17].

We observed a predominant upregulation of proinflammatory genes, like *IL-1beta*, *IL-6*, *iNOS*, and *TNF-alpha* and anti-inflammatory genes, like *Arg1*, *CCL11*, *IL-1*, and *TGF-beta* immediately after LPS sensitization. We demonstrate strong microglia activation and proinflammatory gene expression that exacerbates its reaction after a second insult (like HI). Anti-inflammatory genes showed minor reactions over the times analyzed. Interestingly, HI alone showed a delay in the proinflammatory expression pattern compared to LPS/HI, while anti-inflammatory cytokine expression remained unchanged. Microglia are immune cells which permanently reside in the CNS [25]. Following CNS pathologies, microglia activation happens as a first immune response for the removal of threatening compounds [28, 29, 61, 68, 69]. However, microglia responses often fail in the removal of threats or even result in an escalating effect of a vicious cycle of unresolved local cytotoxic inflammation, which might override the beneficial effect of these cells [36, 51, 53, 61, 70].

Proinflammatory cytokines like IL-1beta, IL-6, IL-12, iNOS, and TNF-alpha are likely one of the first immune mediators which show an upregulation just after an injury. They strongly enhance inflammatory responses and have profound effects on blood brain barrier (BBB) permeability, cell death via programed necrosis, glial activation, immune cell recruitment, neuron excitotoxicity damage, and ultimately in neurodegeneration [51, 71–81]. It has been demonstrated that neuronal self-injury after exposure to LPS could be alleviated by using specific cytokine blockers, as it was already shown for IL-1beta [78]. On the other side, some cytokines like IL-6 has a dual function, where high levels early after HI insults are associated with adverse HIE outcomes; a secondary peak later after HIE was associated with better outcomes by regulating expression levels of IL-1beta and TNF-alpha [56, 82–85] and by its ability to promote neuronal differentiation of neural stem progenitor cells (NSPCs) and promote angiogenesis [86].

The most intensively studied inflammasome is NLRP-3, and it has been shown to be involved in many neurological diseases in adults [87]. NLRP-3 inflammasome mediates Caspase-1 activation in response to cellular damage, with consequently activation and secretion of proinflammatory cytokines like IL-1beta and IL-18 [88, 89]. Stimuli like LPS/HI trigger inflammasome assembly and activation of pyroptosis, a form of cell death [78, 90, 91]. It has previously been demonstrated that after inhibition of NLRP-3, using MCC950 inhibitor, there is a reduction of pyroptosis in injured rat brain with HIE [92]. After infection/inflammation and HI, regulatory pathway involvement might explain how LPS preexposure significantly increases the vulnerability of the newborn brain to a mild hypoxic-ischemic event [89, 92, 93].

While our study shows a pronounced proinflammatory expression and the activation of NLRP-3 inflammasome, anti-inflammatory cytokines did not show a major upregulation over the time. Only after LPS sensitization, a high increase in the anti-inflammatory gene expression was observed, except for *CD206* and *LIF*. However, after HI, *CCL11*, *IL-4*, *LIF*, and *TGF-beta* showed a continued upregulation that did not last over time for all the treatments. Anti-inflammatory cytokines regulate the differentiation of specific T-cells (TH17) [94], maturation and normal homeostasis of microglia cells [95], detoxification by removing excessive nitrogen [96, 97], promotion of neurogenesis, and axonal repair mechanisms [98]. Interestingly, our RNA sequence data point out other cytokines regulated after LPS/HI that gained increased interest over the past years due to their anti-inflammatory function. It has been demonstrated in a neonatal mouse model of cerebral ischemia, CCL11 levels are also upregulated after cerebral ischemia, which results in promoting migration of NSPCs in these mice [99, 100]. Another interesting cytokine was Leukemia Inhibitory Factor (LIF), where it has been shown in a neonatal mouse HI model, a reduction of astrogliosis and microgliosis after intranasal LIF administration, showing preservation of myelin [48].

We clearly observed in our study that a single LPS dose triggers a cascade of inflammatory responses, presensitizing the brain to a major inflammatory state, making it vulnerable to a second insult compared to HI alone. TH has become the standard treatment for neonates with HIE. However, the preexistence of an inflammatory status before HI could reduce the therapeutic window for optimal neuroprotection after TH and could answer why neonates with presensitized brain injury do not benefit from the treatment. We show with our results a time window for the activation of microglia and secretion of different cytokines before and after HI and how a presensitized brain increases the activation of microglia cells into a predominantly M1 phenotype.

The transition between M1/M2 microglia could potentially lead to novel treatment options and/or improve the actual TH. Depletion of anti-inflammatory cytokines has shown impaired oligodendrocytes maturation and subsequent hypomyelination of gray matter tracts as well as postnatal loss of cortical interneurons [101], while enhancing their secretion promotes the phagocytic activity and migration of microglia cells through apoptotic cells, explaining the relevant function of early expression in the clearing of dead cells after an insult [102, 103].

Our study presents some limitations. We could not differentiate between gender and individual subjects because of the technique chosen to isolate microglia. However, we randomized each group between genders, and each group had an equal amount of animals with the same gender. However, further studies are needed to understand the differences between genders in this model. Another limitation is that we have analyzed gene expression patterns and not protein levels. This remarks the importance of further experiments at the protein level in order to confirm our results.

Understanding the role of cytokines in the evolution of neonatal brain injury, as well as the dynamic nature of cytokine release after a hypoxic-ischemic insult, is a promising avenue for identifying biomarkers of ongoing brain injury in newborns with antenatal/postnatal infection/inflammation following HI, especially those that do not show an improved outcome after TH [84, 104–107].

The actual challenge in neonatal asphyxia is to find the line between the beneficial aspects of neuroinflammation following the insult to allow neuroprotection and regeneration while at the same time minimizing its harmful effects in the newborn CNS. Identified key factors that could be used for an early identification of infants with an extensive and potentially damaging neuroinflammatory response of those with moderate inflammation would present new options for a more individualized therapeutic approach in neonatal asphyxia as well as determine the impact of TH and/or to find other neuroprotective treatments, with a potential improvement in future clinical studies that will help to further improve outcome in asphyxiated newborns, especially in countries with high perinatal infection and perinatal asphyxia rates.

## 5. Conclusion

Our results demonstrate that microglia are early key mediators of the inflammatory response and inflammation sensitization exacerbates the inflammatory response following HI, polarizing into a predominant proinflammatory M1 phenotype in the early hours post HI. This may explain why antenatal/postnatal infection-/inflammation-correlated HIE shows an unfavorable outcome compared to HIE alone and that cooling is not beneficial in the context of inflammation-sensitized HIE. Additionally, we demonstrate the involvement of the NLRP3 inflammasome, highlighting one potential regulatory pathway in our model. These findings will help us to better understand the complex inflammatory mechanisms and could be the start point to study microglia polarization to a more beneficial M2 phenotype at specific time points during the insult. However, more research in the topic is needed in the molecular mechanism that gives us in the future the possibility to early intervene and offer new treatment options that will help to further improve outcome in asphyxiated newborns, especially in developing countries with higher infection rates.

## Figures and Tables

**Table 1:** Primer list used for the real-time PCR.

Gene	Official symbol	Entrez gene ID	Product number
Arg1	Arg1	29221	Rn00691090_m1
*β*-Actin	Actb	81822	Rn00667869_m1
CCL11	Ccl11	29397	Rn00569995_m1
Caspase 1	Casp1	25166	Rn00562724_m1
CD206	Mrc1	291327	Rn01487342_m1
IL1beta	Il1b	24494	Rn00580432_m1
IL4	Il4	287287	Rn99999010_m1
IL6	Il6	24498	Rn01410330_m1
IL18	Il18	29197	Rn01422083_m1
IL12	Il12a	84405	Rn00584538_m1
iNOS	Nos2	24599	Rn00561646_m1
LIF	Lif	60584	Rn00573491_g1
NLRP3	Nlrp3	287362	Rn04244621_m1
TGF-beta	Tgfb1	59086	Rn00572010_m1
TNF-alpha	Tnf	24835	Rn01525859_g1

## Data Availability

The raw data supporting the conclusion of this article will be made available by the authors, without undue reservation, to any qualified researcher.
